# Physiotherapy students’ trust in social-media physiotherapy influencers: implications for digital-literacy training in medical education

**DOI:** 10.1186/s12909-025-07760-0

**Published:** 2025-08-28

**Authors:** Bartosz Wilczyński, Hubert Antczak, Karol de Tillier, Marcin Taraszkiewicz, Krzysztof Sobczak³, Katarzyna Zorena

**Affiliations:** 1 Department of Immunobiology and Environment Microbiology, Medical University of Gdansk, Gdańsk, Poland; 2 Gdansk College of Health, Gdańsk, Poland; 3 ³Department of Sociology of Medicine and Medical Communication, Medical University of Gdańsk, Gdańsk, Poland

**Keywords:** Physiotherapy students, Health influencers, Social media, Digital literacy, Medical education, Curriculum design

## Abstract

**Background:**

Health-professional students increasingly learn via social-media influencers, yet the factors that make these sources trustworthy are unknown. Understanding this is critical for designing effective digital-literacy curricula. We investigated physiotherapy students’ behavioural (following, purchasing) and attitudinal (trust) responses to social-media physiotherapy influencers and identified factors associated with higher trust.

**Methods:**

We conducted a cross-sectional, computer-assisted web survey of 314 physiotherapy students from Polish universities​​. The questionnaire captured demographics, social-media use, influencer engagement, critical-appraisal training and attitudes toward influencer content. We used ordinal logistic regression to examine how demographic, behavioural, and attitudinal variables predicted students’ trust in physiotherapy influencers.

**Results:**

Most respondents (77.4%) followed at least one physiotherapy influencer and 46% had bought products they endorsed​​. Overall, 61% expressed high trust​​. Trust in influencers was most strongly predicted by frequent information-seeking from influencers (OR = 3.54, 95% CI 2.45–5.22), perceiving them as more informative than academic staff (OR = 2.00, 95% CI 1.46–2.76), and intensive Instagram use (OR = 1.41, 95% CI 1.06–1.87). In contrast, age, study year, and prior critical-appraisal training were not significant predictors of trust. Although 62% acknowledged commercial bias, these students still reported high trust and continued engagement, revealing cognitive dissonance.

**Conclusions:**

Physiotherapy students trust social-media influencers for professional knowledge, with platform use and perceived informativeness outweighing formal training in shaping this trust. To address this disconnect, medical-education programmes should move beyond traditional critical appraisal and embed authentic, influencer-based digital-literacy exercises that reflect students’ real-world media habits and tackle credibility cues and commercial persuasion.

**Supplementary Information:**

The online version contains supplementary material available at 10.1186/s12909-025-07760-0.

## Background

Social media has become a dominant source of health-related information for students in the health professions, including physiotherapy [1–3]. While social media platforms can facilitate access to up-to-date, evidence-based content [3], they also host unregulated information of highly variable quality [1, 3, 4]. In musculoskeletal care, for example, treatment preferences often diverge from clinical guidelines, with unsupported or promotional interventions preferred over those with proven efficacy [5]. This raises questions about the sources shaping students’ knowledge and may ultimately undermine evidence-based patient care and safety. If left unaddressed, these patterns may contribute to the normalisation of low-value care.

A significant portion of online content is produced by so-called “health influencers”—social media figures who blend personal narratives, health advice, and commercial endorsements [6]. Ernst described this phenomenon as *celebrity-based medicine*, where influence derives more from visibility than from clinical authority [7]. Influencer messaging often relies on emotional appeal, perceived authenticity, and follower closeness to establish trust [5, 8, 9]. Some students may view these figures as more relatable—and thus potentially more credible—than academic professionals, although empirical data are scarce [10].

The marketing power of influencers lies not only in their reach but also in how they frame health behaviours as pathways to personal success, beauty, or productivity [9]. This framing can shape students’ expectations of medical practice, subtly redefining what counts as credible advice [11, 12]. While such content may enhance engagement, it also risks promoting over-simplified, biased, or commercially driven models of care [6]. For physiotherapy education—where evidence-based reasoning and patient-centred care are core values—this influence presents both pedagogic challenges and opportunities for curricular innovation in physiotherapy education [5].

Trust plays a central role in this dynamic. Students must learn to evaluate competing information sources and develop a professional identity grounded in critical thinking [13]. Yet, the extent to which current educational strategies prepare students to navigate influencer-rich environments remains unclear [12]. Prior research has documented barriers to implementing evidence-based physiotherapy, including time constraints, patient demands, and information overload [14], but little attention has been paid to the influence of digital trust and social media behaviour on these barriers. Evidence suggests that social media use can support self-regulated learning among medical students [15], but this potential depends on students’ ability to critically appraise what they encounter [16]. It remains unknown whether physiotherapy students apply critical reasoning to influencer content, or whether trust persists even when bias is recognised—a phenomenon that could undermine evidence-based decision-making and reshape clinical norms [17]. Addressing this issue is essential as students increasingly blend formal and informal learning sources to construct their professional knowledge [18].

For the purposes of this study, we defined a *physiotherapy influencer* as: *“An individual who gains popularity on social media by promoting physiotherapy-related content*,* healthy lifestyles*,* or general well-being. These individuals achieve high visibility and influence*,* often being perceived as authorities on health and body care by their followers”.* This construct was developed by the authors based on literature review and pilot feedback. While the term *health influencer* is widely used in the literature [19, 20], we focus specifically on social media figures whose content relates to physiotherapy. Therefore, we use the term *physiotherapy influencer* throughout this paper. Although the questionnaire used the term ‘health celebrity’ for comprehension purposes, we treat it as equivalent to ‘physiotherapy influencer’ and use the latter consistently throughout the manuscript. This study addresses a gap in the literature by exploring how physiotherapy students engage with social media physiotherapy influencers. Specifically, we aimed to: (1) describe behaviours such as following, trusting, and purchasing based on influencer recommendations; (2) assess students’ attitudes, perceived credibility, and exposure to critical appraisal training; and (3) identify predictors of trust using behavioural, attitudinal, and platform-related variables. We also explored differences between full-time and part-time students to examine whether patterns of engagement and trust varied by study mode. Findings may inform the design of more context-relevant digital-literacy strategies within health professions education.

## Methods

### Study design

The study was conducted as a cross-sectional, web-based survey followed the principles of the Declaration of Helsinki and the European GDPR regulations applicable to research involving human participants. The study followed the CHERRIES checklist for online surveys (see Additional File 1). Participation was voluntary and anonymous. Informed consent was obtained from all adult students prior to data collection (and included: purpose of the study, principal investigator, expected time commitment, anonymity).

### Questionnaire development

The questionnaire was developed by an interdisciplinary research team: physiotherapists, lecturers, medical sociologists, and physiotherapy students. Initial survey items were drafted based on a review of the literature on social media use in health communication, digital trust, and influencer credibility. Two consultation rounds were held with specialists in medical sociology and communication (K.S.), physiotherapists (B.W., K.de T.) and student co-developers (H.A., M.T.) to finalize the terminology and structure.

A pilot test with 32 students from two universities helped assess clarity, response burden, and content validity. Revisions followed based on feedback from focus groups and pilot respondents.

### Participants and recruitment

The study employed a convenience sampling strategy. Eligible participants were physiotherapy students enrolled in full-time or part-time programmes at any Polish university. Inclusion criteria were: (1) sufficient Polish language proficiency (inferred from enrolment in Polish-language physiotherapy programmes and completion of the Polish-language survey), and (2) regular social media use, defined as accessing at least one platform (Instagram, Facebook, TikTok, YouTube, or X/Twitter) for physiotherapy-related content on a daily basis. Additional inclusion criteria included provision of informed consent and complete responses to key variables related to social media use (e.g. minutes per day, hours per week). Twelve incomplete responses were excluded.

Participants were recruited between 13 December 2023 to 10 May 2024 using a targeted digital strategy. Recruitment channels included physiotherapy student groups on Facebook, Instagram, TikTok messages, online forums, institutional email lists, and official university communication.

### Survey instrument

The final version of the questionnaire included adaptive questioning, 11 closed-ended questions, 4 open-ended questions, and 11 Likert-scale items (1 = strongly disagree to 5 = strongly agree). The questionnaire was administered using a one-question-per-page format, with each item appearing on a separate screen. The full survey consisted of 17 screens. This design was chosen to minimize cognitive load and encourage higher completion rates by allowing participants to focus on one question at a time. The full survey is available in Additional file 2. Before responding to relevant items, participants were presented with the definition of a *‘Physiotherapy Influencer*.’ In the questionnaire, the phrase *‘Health Celebrity*’ was used in the context of physiotherapy to maximise respondent comprehension. For the purposes of analysis and interpretation, the terms *‘Physiotherapy Influencer*’ and *‘Health Celebrity*’ were treated as equivalent. The instrument comprised five domains:

1. *Demographics*: age, gender, year of study (1–5), study mode (full-time/part-time), university affiliation.
2. *Social Media Usage*: average daily and weekly time spent on social media for professional/research purposes, and frequency of using specific platforms (Instagram, TikTok, YouTube, Facebook, X) to follow physiotherapy influencers (rated on a 5-point scale from “never” to “very often”).
3. *Influencer Engagement and Behaviour*: regular observation of at least one influencer (yes/no), number of influencers followed, prior purchasing behaviour based on influencer recommendations, and willingness to invite an influencer to teach at university.
4. *Trust and Attitudinal Construct*s: this domain assessed students’ perceived credibility, reliability, and informational value of physiotherapy influencers, collectively referred to as *trust*. These dimensions were chosen to reflect the perceived believability and usefulness of influencer content in a professional context. Trust was operationalised using a single item: “*Physiotherapy Influencers are a valuable and reliable source of information for me*” rated on a 5-point Likert scale from 1 (strongly disagree) to 5 (strongly agree). Additional items measured perceived informativeness compared to academic staff (*“Physiotherapy Influencers*,* in my opinion*,* provide more valuable knowledge than academic teachers during lectures/seminars/exercises”*), frequency of information-seeking, perceived commercial bias, and support for content regulation by national institutions (e.g. Polish Chambers of Physiotherapists).
5. *Critical Appraisal Training*: whether students had been taught to evaluate the credibility of online information sources as part of their physiotherapy education. Critical appraisal exposure was assessed using one item: *“As part of my physiotherapy education*,* I was taught how to assess credibility and critically evaluate information sources from social media platforms*,*”* also rated on a 5-point Likert scale (1 = strongly disagree, 5 = strongly agree).

### Data collection procedures

Data were collected through the CAWI (Computer-Assisted Web Interview) technique. To minimize missing data and prevent “faming” (i.e., multiple submissions by the same respondent), we used a professional online well-protected research service (www.eBadania.pl), which automatically blocked IP addresses from which a questionnaire had already been submitted.

### Statistical analysis

All analyses were conducted using R version 4.3.2 (R Core Team, 2023) with the MASS package. Descriptive statistics were used to summarize demographics, social media usage, influencer engagement, and trust variables. Group comparisons between full-time and part-time students were performed using tests χ² Pearson for categorical variables and Mann–Whitney U tests for non-normally distributed continuous variables. To identify factors associated with trust in physiotherapy influencers, we performed an ordinal logistic regression. The outcome variable (trust) was derived from a 5-point Likert-scale item: “*Physiotherapy Influencers are a valuable and reliable source of information for me*”. Independent variables included: Demographic characteristics (age, gender, year of study), social media usage (platform-specific frequency, time on professional content), attitudinal predictors (perceived information acquisition, critical appraisal training, and perception of influencers’ value compared to academic staff).

Odds ratios (ORs) and 95% confidence intervals (CIs) were reported. The Brant test was used to assess the proportional odds assumption. A significant violation was detected for the *gets info from influencers* variable (χ² = 13.82, df = 3, *p* < 0.001). Although alternative modeling strategies (e.g., partial proportional odds models) were considered, convergence issues limited their feasibility. We acknowledge that interpretation of the affected variable (‘*gets information from influencers*’) should be treated cautiously due to non-proportionality. Although we retained this variable in the main model to preserve comparability and interpretability, we also explored trust distribution across response levels to contextualize this violation (see Supplemental Table 1).

Therefore, we present the full model using standard proportional odds regression, supported by stratified descriptive analyses to contextualize the assumption violation. Secondary analyses explored whether critical appraisal training was associated with trust, skepticism, or influencer-related behaviour. We used Spearman’s rank correlations for ordinal variables and χ² tests for categorical outcomes. Correlations were interpreted using Cohen’s benchmarks, with *p* < 0.05 considered statistically significant. We also examined the relationship between time spent on social media (both daily and weekly use) and behavioural outcomes using Spearman’s rho. To explore potential cognitive dissonance, we tested whether perceived influencer bias was associated with continued engagement (*gets info from influencers*), trust (*trust influencers*), or purchasing behaviour (*bought product*), using two-tailed tests with significance set at *p* < 0.05. No a priori power calculation was conducted; however, the final sample (*N* = 314) meets recommended thresholds for ordinal logistic regression models with multiple predictors (minimum 10–20 cases per variable), allowing for adequate model stability and generalizability.

### Analytical strategy

Each of the three study objectives was addressed using a corresponding statistical approach. Objective 1 (describing behavioural patterns such as following, purchasing, and information-seeking) was addressed using descriptive statistics. Objective 2 (examining students’ attitudes, perceived credibility, and exposure to critical appraisal training) was addressed through item-level summaries and subgroup comparisons (full-time vs. part-time) using χ² and Mann–Whitney U tests. Objective 3 (identifying predictors of trust) was addressed using ordinal logistic regression, with trust as the outcome variable and demographic, behavioural, and attitudinal predictors. Additional exploratory analyses included pairwise Spearman correlations and stratified trust distributions to contextualise regression findings and model assumptions.

## Results

### Sample characteristics

A total of 314 physiotherapy students from 32 universities across Poland completed the survey. Twelve responses were excluded due to missing data. The sample consisted of 239 full-time (76.1%) and 75 part-time (23.9%) students (Table 1). The mean age was 22.7 years (SD = 3.7), with part-time students significantly older than full-time students (U = 5355.5, *p* < 0.001). Gender distribution did not differ significantly between groups (χ²(2) = 2.64, *p* = 0.2671), but year of study did (χ²(4) = 27.39, *p* < 0.001), with most part-time students in Year 2 (61.3%). The use of social media for physiotherapy content did not differ significantly between groups in terms of reported daily time (*p* > 0.05). The largest proportion of participants came from the Gdańsk College of Health (46.6%), followed by the University School of Physical Education in Kraków (9.9%), the Academy of Physical Education in Warsaw (7.6%), and the Medical University of Gdańsk (6.1%). Table 1 also reports students’ self-estimated daily and weekly time spent on social media specifically for following physiotherapy influencers and consuming related professional content.

**Table 1:** Demographic and social media usage characteristics of physiotherapy students by study mode

	Overall	Full-time	Part-time
Participants	*N* = 314	*n* = 239 (76.1%)	*n* = 75 (23.9%)
Age (years)	22.7 (3.7)	21.9 (1.7)	25.2 (6.3)
Mean (SD), Range	18.0–49.0	18.0–29.0	20.0–49.0
Gender			
Female	226 (72.0%)	177 (74.1%)	49 (65.3%)
Male	87 (27.7%)	61 (25.5%)	26 (34.7%)
Refused to answer	1 (0.3%)	1 (0.4%)	0 (0.0%)
Year of study			
Year 1	27 (8.6%)	27 (11.3%)	0 (0.0%)
Year 2	124 (39.5%)	78 (32.6%)	46 (61.3%)
Year 3	41 (13.1%)	36 (15.1%)	5 (6.7%)
Year 4	50 (15.9%)	44 (18.4%)	6 (8.0%)
Year 5	72 (22.9%)	54 (22.6%)	18 (24.0%)
Time on social media to follow Physiotherapy Influencers			
Minutes per day mean (SD) median (IQR)	49.8 (54.0) 30.0 (40.0)	52.6 (59.0) 30.0 (40.0	40.9 (32.0) 30.0 (42.5)
Hours per week mean (SD) median (IQR)	4.6 (4.5) 3.5 (3.0)	4.6 (4.6) 3.5 (3.0)	4.8 (4.2) 4.0 (2.2)

Legend. Gender: χ²(2) = 2.64, *p* = 0.2671 → no significant difference, Year of Study: χ²(4) = 27.39, *p* < 0.001 → significant difference, Age: Mann–Whitney U = 5355.5, *p* < 0.001 → significant difference, Minutes/day: Mann–Whitney U, *p* = 0.5523, Hours/week: Mann–Whitney U, *p* = 0.3363

### Exposure and Platform Preferences

**Fig. 1:**
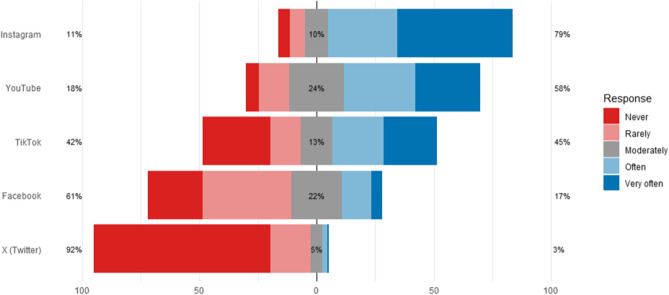
Use of Social Media Platforms to Follow Physiotherapy Influencers. Legend. Students reported the highest engagement with Instagram, with 49.0% (*n* = 154) indicating they “very often” follow physiotherapy influencers on the platform, and an additional 29.6% (*n* = 93) selecting “often”. YouTube also showed substantial usage, with 27.7% (*n* = 87) reporting “very often” and 30.3% (*n* = 95) “often”. TikTok followed closely, with 22.6% (*n* = 71) indicating “very often” and 22.0% (*n* = 69) “often.”. In contrast, engagement with more traditional or text-based platforms was notably lower. On Facebook, 23.2% (*n* = 73) reported “never” using the platform to follow physiotherapy influencers, and only 4.8% (*n* = 15) did so “very often”. The lowest engagement was observed on X (formerly Twitter), where 75.2% (*n* = 236) reported “never” using the platform for this purpose, and only 0.6% (*n* = 2) selected “very often”

Students reported the highest engagement with physiotherapy content on Instagram, with 49.0% reporting “very often” and 29.6% “often” following physiotherapy influencers (Fig. 1). YouTube was next (27.7% “very often”, 30.3% “often”), followed by TikTok. Facebook and X/Twitter showed minimal use, with 75.2% of students reporting they “never” follow physiotherapy influencers on X.

### Influencer engagement and behavioural impact

Most participants (77.4%, *n* = 243) reported regularly observing at least one physiotherapy influencer on social media. Nearly half of the students (44.4%) followed more than five influencers, and an additional 41.6% followed between three and five, and 14.0% followed 1–2. Purchasing behaviour was also common, with 45.7% (23.9% more than once, 21.8% once) having bought training aids, clothing, or other products recommended by influencers, including 23.9% who did so more than once.​​.

### Trust and attitudinal measures

**Fig. 2:**
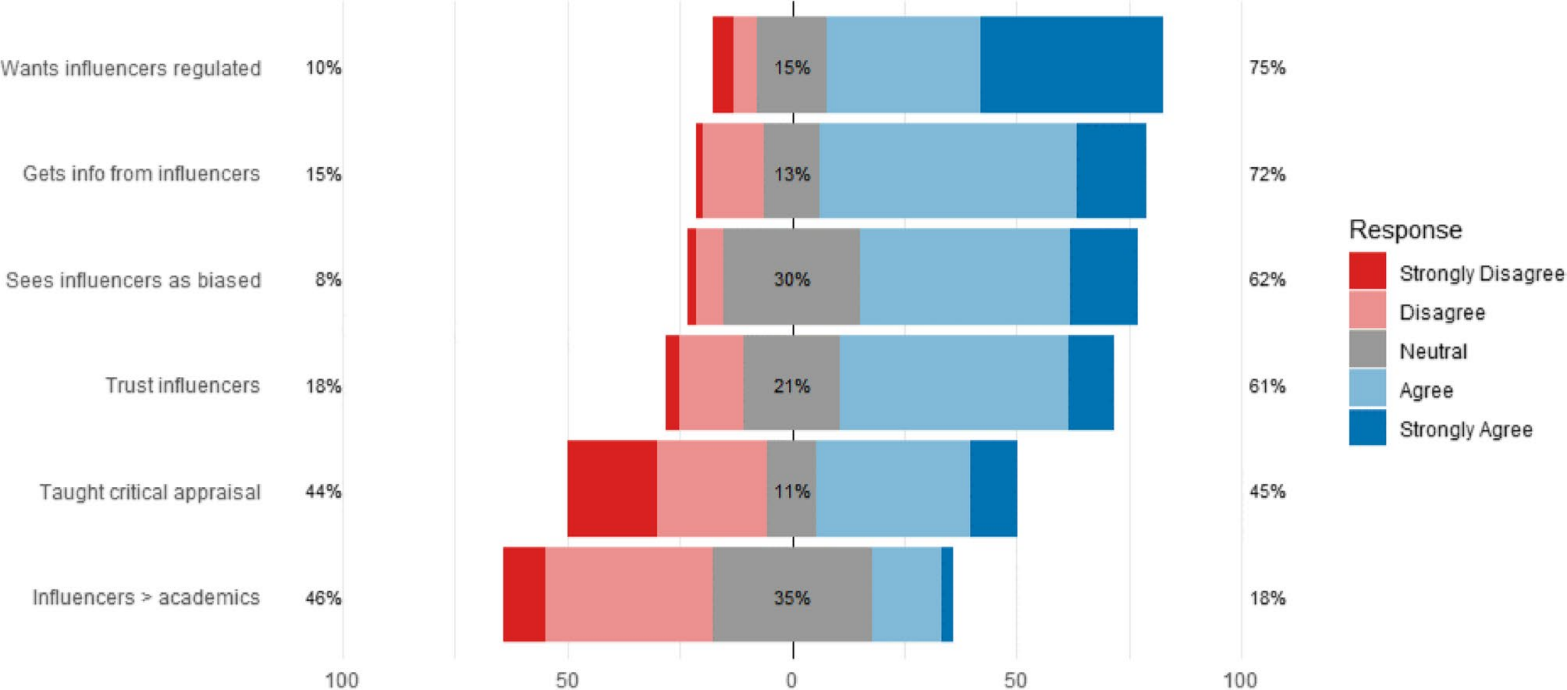
Student Attitudes Toward Physiotherapy Influencers. Legend. Stacked bar chart showing Likert-scale responses (1= Strongly Disagree to 5 = Strongly Agree) for six key attitudinal statements. Responses reveal cognitive dissonance: despite recognising bias, students report high trust in influencers and strong support for their inclusion in education and regulation by authorities

Figure 2 visualises students’ attitudes across six dimensions, revealing a notable paradox: although 62% of participants perceived physiotherapy influencers as commercially biased, 61% still reported high trust, and 75% supported their involvement in education. This pattern underscores the cognitive dissonance observed in our findings—students simultaneously recognise bias yet continue to view influencers as credible information sources.

Participants generally expressed moderate to high trust in physiotherapy influencers (Fig. 2), 50.6% (*n* = 159) agreed and 10.5% (*n* = 33) strongly agreed that influencers are a valuable and reliable source of information, while only 3.2% (*n* = 10) strongly disagreed. Similarly, 57.0% (*n* = 179) agreed and 15.3% (*n* = 48) strongly agreed that they often obtain valuable physiotherapy information from influencers. Regarding critical appraisal training, responses were more divided. 34.1% (*n* = 107) agreed and 10.5% (*n* = 33) strongly agreed that they had been taught to critically evaluate information from social media. In contrast, 24.2% (*n* = 76) disagreed and 20.1% (*n* = 63) strongly disagreed, with another 11.1% (*n* = 35) expressing no clear opinion—highlighting a substantial gap in formal media literacy training.

When comparing the value of information from influencers to that of academic staff, 36.9% (*n* = 116) disagreed and 9.6% (*n* = 30) strongly disagreed that influencers provide more valuable knowledge. Only 2.5% (*n* = 8) strongly agreed.

Perceptions of bias were high: 46.8% (*n* = 147) agreed and 15.0% (*n* = 47) strongly agreed that physiotherapy influencers are generally biased toward specific products or services.

Finally, 74.8% of participants supported regulation: 34.4% (*n* = 108) agreed and 40.4% (*n* = 127) strongly agreed that influencer content should be monitored by national institutions to ensure safety and scientific accuracy.

### Trust Predictors and converging patterns

An ordinal logistic regression identified three significant predictors of trust in physiotherapy influencers (Table 2): frequency of obtaining information from influencers (OR = 3.54, 95% CI [2.45, 5.22], *p* < 0.001), perceiving influencers as more informative than academic staff (OR = 2.00, 95% CI [1.46, 2.76], *p* < 0.001), and Instagram use (OR = 1.41, 95% CI [1.06, 1.87], *p* = 0.018).

The Brant test indicated that the proportional odds assumption held for the overall model (χ² = 7.86, df = 27, *p* > 0.999). However, a violation was detected at the individual predictor level for one predictor *“gets information from influencers”* (χ² = 13.82, df = 3, *p* < 0.001), suggesting that its effect on trust may not be constant across all outcome levels.

These associations were supported by moderate-to-strong Spearman correlations with trust (*r* = 0.52, 0.37, and 0.31, respectively; see Supplemental Table 2) and were further reinforced in stratified analyses. For example, students who frequently obtained information from influencers had a mean trust score of 4.09, compared to 3.12 among those with lower exposure (see Supplemental Table 1). These converging results suggest a robust and consistent relationship between digital engagement patterns and trust formation, regardless of an analytic approach. Other predictors, including platform usage outside Instagram, age, study year, and critical appraisal training, were not significantly associated with trust (*p* > 0.05).

**Table 2:** Ordinal logistic regression predicting trust in physiotherapy influencers

Predictor	Coefficient (β)	Standard Error	*p*	Odds Ratio	95% CI
Instagram use	0.34	0.15	0.018*	1.41	[1.06, 1.87]
YouTube use	0.10	0.12	0.420	1.10	[0.87, 1.39]
Facebook use	0.05	0.12	0.659	1.06	[0.83, 1.34]
TikTok use	−0.05	0.09	0.581	0.95	[0.79, 1.14]
Minutes per day	0.001	0.003	0.744	1.00	[1.00, 1.01]
Gets info from influencers	1.27	0.19	< 0.001***	3.54	[2.45, 5.22]
Critical appraisal taught	0.08	0.10	0.454	1.08	[0.88, 1.32]
Thinks influencers better than academics	0.69	0.16	< 0.001***	2.00	[1.46, 2.76]
Age	−0.04	0.04	0.279	0.96	[0.89, 1.03]

Legend. *p < 0.05, **p < 0.01, ***p < 0.001. *CI* Confidence Interval. The dependent variable is trust in physiotherapy influencers measured on a 5-point Likert scale (1 = Strongly disagree to 5 = Strongly agree). The proportional odds assumption was tested using the Brant test, which indicated a violation for the ‘gets info from influencers’ variable (χ² = 13.82, df = 3, *p* < 0.001).

### Critical appraisal and perceived bias

We examined whether students with stronger critical appraisal training differed in trust, skepticism, or behaviour. No significant association was found between critical appraisal and trust in influencers (ρ = –0.025, *p* = 0.656) or product purchasing (ρ = –0.048, *p* = 0.460). However, students who reported greater exposure to critical appraisal training were slightly more likely to perceive influencers as biased (ρ = –0.125, *p* = 0.027), suggesting a weak association between training exposure and scepticism. This increased scepticism did not translate into reduced trust or disengagement.

### Cognitive dissonance patterns

Despite recognising bias, students continued to engage with influencers. Among those who perceived physiotherapy influencers as biased (61.8%), the mean trust score remained high (M = 4.09). Perceived bias showed no significant correlation with trust (ρ = 0.057, *p* = 0.310) or product purchasing behaviour (ρ = − 0.058, *p* = 0.369). However, it was weakly but significantly associated with information-seeking behaviour (ρ = 0.186, *p* < 0.001), indicating that students who acknowledged influencer bias were still likely to obtain information from them. These findings reflect a cognitive dissonance pattern—where skepticism does not reduce engagement or behavioural susceptibility.

### Group differences: full-time vs. part-time

A subgroup comparison (Table 3) found minimal differences between full-time and part-time students in trust levels, engagement, or attitudes. The only significant difference was in critical appraisal training: 78% of part-time students reported receiving such training, compared to 60% of full-time students (χ² = 6.13, *p* = 0.013, Cramér’s V = 0.159). No significant group differences were found in trust toward influencers, bias perception, purchasing behaviour, or support for regulation.

**Table 3:** Comparison of full-time vs. part-time students on agreement (Likert 4–5 or “Yes”) for key variables

	Full-time		Part-time				
Factor	Agree/Strongly Agree (%)	Other (%)	Agree/Strongly Agre (%)	Other (%)	Chi²	*p*	Cramér’s V
Trust influencers	124 (68%)	59 (32%)	44 (73%)	16 (27%)	0.42	0.516	0.042
Gets informations from influencers	145 (79%)	38 (21%)	50 (83%)	10 (17%)	0.26	0.613	0.032
Critical appraisal taught	109 (60%)	74 (40%)	47 (78%)	13 (22%)	6.13	0.013*	0.159
Thinks influencers better than academics	41 (22%)	142 (78%)	21 (35%)	39 (65%)	3.14	0.076	0.114
Sees influencers as biased	115 (63%)	68 (37%)	38 (63%)	22 (37%)	0.00	> 0.999	0.000
Wants influencers regulated	146 (80%)	37 (20%)	48 (80%)	12 (20%)	0.00	> 0.999	0.000
Wants influencers to teach	153 (84%)	30 (16%)	45 (75%)	15 (25%)	1.68	0.194	0.083
Bought product from influencers	38 (21%)	145 (79%)	20 (33%)	40 (67%)	3.27	0.071	0.116

Legend: *p < 0.05.

## Discussion

### Summary of key findings

This study examined trust in physiotherapy influencers among students and identified key behavioural and perceptual predictors. Information-seeking behaviour, platform usage, and the perception that influencers offer more value than academic staff were the strongest predictors of trust. Notably, critical appraisal training was not associated with lower trust or reduced engagement, and students continued to follow influencers despite recognising bias—indicating a form of cognitive dissonance.

### Interpretation and implications for medical education

Polish physiotherapy education operates within a nationally standardized system, regulated by the Polish Accreditation Committee (PAC), a member of the European Consortium for Accreditation in Higher Education (ECA), International Network for Quality Assurance Agencies in Higher Education (INQAAHE), and European Association for Quality Assurance in Higher Education (ENQA). PAC evaluations are mandatory and directly affect institutional licensing and admissions [21]. Curricula across universities follow structured models with a strong emphasis on professional standards and national licensure requirements. This regulated context ensures a consistent educational framework across institutions, allowing for meaningful cross-university comparisons of student attitudes and behaviours.

Our findings suggest that physiotherapy students are highly active digital learners, who engage extensively with health-related content on social media, particularly via Instagram, YouTube, and TikTok. Over three-quarters of participants reported regularly observing at least one physiotherapy influencer, and nearly half followed more than five. These findings align with prior research showing high social media use for professional purposes among medical students [13]. Similarly, Pilgrim and Bohnet-Joschko [9] and Kaňková et al. [6] highlighted Instagram’s growing role in shaping health-related beliefs, confirming the dominant role of visual platforms among health professions students. Importantly, engagement was associated with behavioural outcomes, with 45.7% reporting purchases influenced by recommendations. Our findings reflect patterns also reported in the fitness and diet domains [9], reinforcing that physiotherapy students may report similar patterns of exposure to persuasive digital content in shaping their health-related beliefs and behaviour.

Our regression analysis showed that for each one-unit increase in Instagram usage, the odds of higher trust increased by 41%, and students who actively obtained information from influencers were over 3.5 times more likely to trust them. Similarly, students who perceived influencers as more informative than academic staff had double the odds of reporting high trust. These findings highlight that trust in our sample was associated with students’ digital habits and perceived utility of influencers. This echoes prior evidence suggesting that physiotherapists often balance clinical evidence with contextual pressures such as patient preferences and peer influence [14]. Similarly, Zadro et al.​ [5] found that many physiotherapy treatment choices deviate from guidelines, underscoring the role of non-academic sources in shaping practice—paralleling our observation that students who valued influencers over academics reported higher trust.

Notably, critical appraisal training was not a significant predictor of trust in our regression model (OR = 1.08, *p* = 0.45), reinforcing the notion that current educational approaches have limited influence on how students evaluate influencer content. This aligns with our finding that 44% of students reported lacking adequate training in evaluating social media content. Qualitative work by O’Connor et al. [22] suggests that in the absence of clear and consistent institutional guidance, students develop informal strategies to navigate content—possibly explaining why appraisal training did not impact trust or behaviour in our sample. Although students with appraisal training were more likely to recognize influencer bias, they did not disengage from such content suggesting that awareness alone does not lead to behavioural restraint. This mirrors findings from implementation science, which show that knowledge alone is insufficient to drive evidence-based practice [14]. The persistence of high trust despite recognized bias illustrates a cognitive dissonance that current curricula in our cohort fail to address. It underscores the need for appraisal training to evolve from abstract principles to applied, emotionally relevant digital media literacy that confronts students’ real-world information habits [9]. Given students’ high levels of social media engagement and sustained trust in influencers despite recognising bias, we propose that digital literacy training should be adapted to include *authentic*,* influencer-based exercises*. These might involve evaluating real social media posts, product endorsements, or educational videos from physiotherapy influencers using evidence-based criteria and facilitated group discussion. Such tasks align more closely with students’ digital environments than traditional critical appraisal training, which typically relies on decontextualised academic articles. Embedding this realism into curricula may help bridge the gap between theoretical knowledge and practical, digital-era decision-making.

There is a clear need to integrate the ability to critically analyse social media content into physiotherapy education itself. In our survey, 44.2% of students indicated that they had not been sufficiently prepared in this area. This gap aligns with our regression and correlational findings, and supports earlier work by Sandars and Schroter [23], who argued that integrating social media into curricula must be paired with skills to critically evaluate digital health content.

These insights carry clear implications for medical education. Educators should rethink how to adapt to a landscape where social media actively shapes student thinking. A recent scoping review of digital assessment in medical education shows that online learning ecosystems now permeate every stage of training and demand new digital competencies from both learners and teachers [24]. Embedding critical digital literacy, influencer analysis, and health communication into curricula seems essential [3, 14, 25]. Our finding that students maintained high trust despite acknowledging bias highlights the importance of confronting real-world content directly within teaching. Training should not only teach students to spot bias but help them develop the reflexive capacity to question why and how they trust non-academic sources, especially when these sources contradict or override institutional teaching [26]. As highlighted by Cutrer et al. [16], the explosion of digital information demands a reorientation in medical education from content delivery to scaffolding students’ ability to critically navigate and apply external information sources as “*Master Adaptive Learners*”. This shift must acknowledge the realities of how modern students learn and where they seek professional guidance [16].

An additional finding of interest was students’ openness to direct engagement with influencers in educational settings. Nearly 79% of respondents expressed willingness to participate in workshops or lectures led by physiotherapy influencers, suggesting a potential avenue for integrating popular communicators into formal education. However, as Cheston et al. [12] emphasize, such integration must be accompanied by rigorous oversight to ensure content accuracy and prevent the spread of unverified information. This aligns with our findings, where 74.8% of students supported regulatory oversight by the National Chamber of Physiotherapists, including 40.4% who strongly agreed and 34.4% who agreed that influencer content should be monitored to ensure safety and scientific validity.

Our cohort also demonstrated high social media engagement (77% followed at least one influencer), but extended previous findings by showing that trust remained high even when bias was acknowledged. Similar surveys confirm how pervasive social-media learning has become. Findyartini et al. linked higher self-regulated-learning scores with more frequent educational use of social platforms among 1,122 Indonesian medical students (low-to-moderate correlations, *r* = 0.20–0.46) [15]. Avci et al. found that 93% of Turkish medical students used social media and 89% did so for professional purposes, yet ethical awareness was weak [13]. These findings suggest that even students with high self-regulated learning may benefit from or respond differently to targeted training on digital persuasion and credibility cues [13, 15].

### Cognitive dissonance and behaviour

Our findings highlight a clear cognitive dissonance in student attitudes toward physiotherapy influencers. While 61.8% of students agreed that influencers are biased toward specific products or services, this recognition did not reduce trust or behavioural engagement. In fact, students who acknowledged bias still reported high trust levels and frequent information-seeking behaviour. This contradiction suggests that students compartmentalize professional skepticism and personal utility—tolerating bias as a trade-off for accessible, actionable content. This may shape professional identity formation, as students appear to construct knowledge frameworks not only on academic rigor, but also on convenience, relatability, and perceived authenticity. Similar patterns have been observed in health communication literature, where perceived trustworthiness often outweighs factual accuracy in shaping health beliefs [27, 28]. For medical educators, this underscores the need to directly address these contradictions in training—not just by teaching evidence appraisal, but by exploring how and why students trust unregulated sources even when they know better.

### Full-Time vs. part-time differences

Although part-time students were significantly more likely to report receiving critical appraisal training (78% vs. 60%), they did not differ meaningfully from full-time students in trust toward influencers, perceived bias, purchasing behaviour, or support for regulation. This suggests that greater exposure to formal training does not necessarily translate into altered engagement with influencer content. One possible explanation is that part-time students’ additional life responsibilities and heterogeneous profiles complicate assumptions linking formal training to behavioural change [29]. Alternatively, institutional differences in curriculum delivery or individual learning goals may account for variations in how appraisal training is internalized, regardless of mode of study. These findings suggest that simply offering critical appraisal instruction is insufficient unless students are supported in applying it meaningfully across learning contexts.

### Strengths and limitations

This study offers novel insights into students’ behavioural and attitudinal interactions with physiotherapy influencers, but several limitations must be acknowledged. First, all data were self-reported, including critical appraisal training, which may not accurately reflect students’ actual skills or competencies. Our measure of critical appraisal was based on self-reported exposure to formal training, which may not reflect actual skill. Some students may have developed appraisal ability through informal or self-directed learning. Therefore, results linking appraisal training to trust should be interpreted with caution. Second, the cross-sectional design and use of ordinal logistic regression preclude any causal interpretations—observed associations cannot establish directionality or underlying mechanisms. The proportional odds assumption was violated for a key predictor (‘gets information from influencers’). Although partial proportional odds models were considered, these did not converge due to sample and model complexity. As a result, the odds ratio for this variable should be interpreted with caution, and future studies should consider non-parametric or alternative ordinal modelling techniques to validate this finding. Third, the sample was skewed, with 46.6% of participants from a single institution, potentially influencing platform usage patterns and exposure norms. The sample was also predominantly female and composed mainly of full-time students. However, this demographic reflects the national physiotherapy workforce and enrolment patterns in Poland, where approximately 74% of physiotherapists are female [30]. Fourth, Instagram emerged as a dominant predictor, while other platforms showed weak or inconsistent associations; future studies should consider clustering or latent class modelling to better capture social media behaviour profiles.

## Conclusion

Physiotherapy students actively engage with social media influencers and often trust them as valuable sources of information, even when aware of potential bias. This trust is associated more with platform behaviour and perceived informativeness than with formal educational exposure to critical appraisal training — suggesting that conventional methods may be insufficient. Medical educators must recognize that students are developing professional attitudes through informal digital channels and respond by embedding practical, context-aware media literacy into curricula. Addressing this disconnect is essential not only for safeguarding evidence-based practice, but also for guiding students in navigating the realities of modern health communication.

## Supplementary Information

Supplementary Material 1.

Supplementary Material 2.

## Data Availability

The data that support the findings of this study are available on request from the corresponding author B.W.
