# Supplementary material for: Physiotherapy students’ trust in social-media physiotherapy influencers: implications for digital-literacy training in medical education
Source: BMC Med Educ. 2025 Aug 28;25:1215. doi: 10.1186/s12909-025-07760-0 (PMC12392578; doi:10.1186/s12909-025-07760-0)
Supplement: Supplementary file 1 — Supplementary Material 1. [file 12909_2025_7760_MOESM1_ESM.docx]

| **Question No.** | **Question** | **Instruction** | **Question Type** | **Min -> Max Answers** | **Description** | **Code** | **Answers** | **Variable Type: Numeric / Text** |
| --- | --- | --- | --- | --- | --- | --- | --- | --- |
| 1 | Do you consent to participate in the research study? | Choose one answer | closed | 1 -> 1 |  | v1 | 1: Yes / 2: No | N |
| 2 | Are you a physiotherapy student? | Select only one answer. | closed | 1 -> 1 |  | v2 | 1: Yes / 2: No | N |
| 3 | Do you use social media? (at least one of: Instagram, Facebook, Twitter, YouTube, TikTok) | Select only one answer. | closed | 1 -> 1 |  | v3 | 1: Yes / 2: No | N |
| 4 | Gender | Select only one answer. | closed | 1 -> 1 |  | v4 | 1: Female / 2: Male / 3: Non-binary / 4: Prefer not to answer | N |
| 5 | Age | Enter a number | open |  |  | v5_t |  | T |
| 6 | Year of study | Select only one answer. | closed | 1 -> 1 |  | v6 | 1: I / 2: II / 3: III / 4: IV / 5: V | N |
| 7 | Study mode | Select only one answer. | closed | 1 -> 1 |  | v7 | 1: Full-time / 2: Part-time | N |
| 8 | University name | Enter the full name of the university | open |  |  | v8_t |  | T |
| 9 | How many minutes per day do you use social media platforms for educational purposes (e.g., following physiotherapy influencers, physiotherapy organizations, learning new methods, checking trends)? | Enter an estimated number of minutes per day | open |  |  | v9_t |  | T |
| 10 | How many hours per week do you use social media platforms for educational purposes (e.g., following physiotherapy influencers, physiotherapy organizations, learning new methods, checking trends)? | Enter an estimated number of hours per week | open |  |  | v10_t |  | T |
| 11 | How often do you use the following social media platforms to observe physiotherapists acting as health celebrities? | Select only one answer. | scale | 1 -> 1 | never / rarely / moderately / often / very often |  |  |  |
|  | Facebook |  |  |  |  | v11_1 | 1: never / 2: rarely / 3: moderately / 4: often / 5: very often | N |
|  | Instagram |  |  |  |  | v11_2 | 1: never / 2: rarely / 3: moderately / 4: often / 5: very often | N |
|  | Twitter |  |  |  |  | v11_3 | 1: never / 2: rarely / 3: moderately / 4: often / 5: very often | N |
|  | TikTok |  |  |  |  | v11_4 | 1: never / 2: rarely / 3: moderately / 4: often / 5: very often | N |
|  | YouTube |  |  |  |  | v11_5 | 1: never / 2: rarely / 3: moderately / 4: often / 5: very often | N |
| 12 | Do you regularly observe the activities of (at least one) physiotherapy health celebrity on social media? | Select only one answer. | closed | 1 -> 1 |  | v12 | 1: Yes / 2: No | N |
| 13 | How many physiotherapy health celebrities do you follow on social media? | Select only one answer. | closed | 1 -> 1 |  | v13 | 1: 1–2 / 2: 3–5 / 3: More than 5 | N |
| 14 | Would you like the health celebrity you observe to teach at your university during lectures/workshops/seminars/exercises? | Select only one answer. | closed | 1 -> 1 |  | v14 | 1: Yes / 2: No / 3: No opinion | N |
| 15 | Have you ever purchased training aids, clothing, or other products recommended by the health celebrity you observe? | Select only one answer. | closed | 1 -> 1 |  | v15 | 1: Yes, more than once / 2: Yes, once / 3: No | N |
| 16 | Please respond to the following statements: | Select one answer for each statement. | scale | 1 -> 1 | Strongly disagree / Disagree / No opinion / Agree / Strongly agree |  |  |  |
|  | Health celebrities are a valuable and reliable source of information for me. |  |  |  |  | v16_1 | 1: Strongly disagree / 2: Disagree / 3: No opinion / 4: Agree / 5: Strongly agree | N |
|  | I often obtain valuable physiotherapy information from health celebrities using social media. |  |  |  |  | v16_2 | 1: Strongly disagree / 2: Disagree / 3: No opinion / 4: Agree / 5: Strongly agree | N |
|  | As part of my physiotherapy education, I was taught how to assess credibility and critically evaluate information sources from social media platforms. |  |  |  |  | v16_3 | 1: Strongly disagree / 2: Disagree / 3: No opinion / 4: Agree / 5: Strongly agree | N |
|  | Health celebrities, in my opinion, provide more valuable knowledge than academic teachers during lectures/seminars/exercises. |  |  |  |  | v16_4 | 1: Strongly disagree / 2: Disagree / 3: No opinion / 4: Agree / 5: Strongly agree | N |
|  | Health celebrities are generally biased toward certain products or services (e.g., selling courses, webinars, physiotherapy products). |  |  |  |  | v16_5 | 1: Strongly disagree / 2: Disagree / 3: No opinion / 4: Agree / 5: Strongly agree | N |
|  | I believe that the content presented by health celebrities should be monitored/verified by national institutions (e.g., KIF) to ensure safety and compliance with scientific knowledge. |  |  |  |  | v16_6 | 1: Strongly disagree / 2: Disagree / 3: No opinion / 4: Agree / 5: Strongly agree | N |
| 17 | Study completed | Thank you for your time | closed | 1 -> 1 |  | v17 | 1: | N |
